# Deferiprone and Iron–Maltol: Forty Years since Their Discovery and Insights into Their Drug Design, Development, Clinical Use and Future Prospects

**DOI:** 10.3390/ijms24054970

**Published:** 2023-03-04

**Authors:** George J. Kontoghiorghes

**Affiliations:** Postgraduate Research Institute of Science, Technology, Environment and Medicine, Limassol 3021, Cyprus; kontoghiorghes.g.j@pri.ac.cy; Tel./Fax: +357-26-272-076

**Keywords:** deferiprone, iron-maltol, ferric maltol, feraccru, accrufer, iron overload, iron deficiency anemia, thalassemia, iron metabolism, iron toxicity, drug design, development and use, orphan drugs, orphan diseases

## Abstract

The historical insights and background of the discovery, development and clinical use of deferiprone (L1) and the maltol–iron complex, which were discovered over 40 years ago, highlight the difficulties, complexities and efforts in general orphan drug development programs originating from academic centers. Deferiprone is widely used for the removal of excess iron in the treatment of iron overload diseases, but also in many other diseases associated with iron toxicity, as well as the modulation of iron metabolism pathways. The maltol–iron complex is a recently approved drug used for increasing iron intake in the treatment of iron deficiency anemia, a condition affecting one-third to one-quarter of the world’s population. Detailed insights into different aspects of drug development associated with L1 and the maltol–iron complex are revealed, including theoretical concepts of invention; drug discovery; new chemical synthesis; in vitro, in vivo and clinical screening; toxicology; pharmacology; and the optimization of dose protocols. The prospects of the application of these two drugs in many other diseases are discussed under the light of competing drugs from other academic and commercial centers and also different regulatory authorities. The underlying scientific and other strategies, as well as the many limitations in the present global scene of pharmaceuticals, are also highlighted, with an emphasis on the priorities for orphan drug and emergency medicine development, including the roles of the academic scientific community, pharmaceutical companies and patient organizations.

## 1. Introduction

Iron is one of the essential metal ions found in all cells of the body, as well as in cancer cells and microbes. It is generally required for many enzymes and other proteins performing vital bodily functions and is involved in many physiological and biological processes, such as oxygen transport, storage and utilization; energy transduction; growth and development; lipid metabolism; and DNA synthesis [1,2,3].

Iron is tightly controlled in the human body using different mechanisms, pathways and proteins, which are involved in its uptake, distribution, utilization, recycling and excretion. Each cell requires and utilizes different amounts of iron and also other essential metal ions such as zinc and copper for different biological functions [1,2,3,4,5,6,7]. In particular, the transfer of iron to all cells of the body is accomplished by transferrin in the blood, and is mediated through transferrin receptors present on the cell membrane, followed by its incorporation in the cells using endocytosis [8,9,10,11]. Once inside the cells, iron is released into a low molecular weight iron pool and used mainly for storage but also for the production of iron-containing enzymes. Iron storage in cells is accomplished by ferritin and hemosiderin. Ferritin contains different amounts of iron, and one molecule of ferritin can store up to 4500 molecules of iron. Hemosiderin is a cluster of ferritin molecules with a broken protein shell, which mainly predominates in iron storage over ferritin in iron overload diseases and also other conditions of focal iron deposition [2,11,12,13,14,15,16].

There is wide variation in the requirements for iron and other metals for different cell types, which in the case of iron is mainly reflected by the number of transferrin receptors on the cell membrane [8,9,10,11,17]. For example, increased transferrin receptors are observed in different types of normal cells such as hepatocytes and erythropoietic cells, and also in certain cancer cell types such as breast, prostate and bladder cancers and in leukemias [8,17,18,19]. The control of the delivery of transferrin iron and also other forms of iron into cells through the modulation of transferrin receptors can directly affect key enzymes and the production of different biomolecules, including metabolites playing important roles in health and disease. Furthermore, the role and function of such metabolites related to iron could also be used for the design of specific drugs and for therapeutic strategies in different diseases [18,19,20].

It is estimated that about 4.5–5.0 g of iron is present in a 70–75 kg normal adult man, which is distributed in the blood and different organs. Most of the iron is in the form of heme in the proteins hemoglobin (2.3–2.6 g) in red blood cells (RBC) and myoglobin (0.32–0.40 g) in muscle. Iron is also distributed in the body, mainly in the form of polynuclear ferric oxyhydroxide phosphate complexes in the iron storage proteins ferritin (0.7 g) and hemosiderin (0.3 g), which are mainly found in the liver, spleen, muscles and bone marrow. Smaller amounts of iron are also found in mitochondrial cytochromes (17 mg), catalase (5 mg), transferrin (4 mg) and non-heme iron-containing enzymes (0.1 g) [20].

At normal physiological conditions and pH levels, iron is mainly found in the ferrous (Fe^2+^) or ferric (Fe^3+^) state forms, and is always bound to different ligands containing oxygen, nitrogen and sulfur [20]. Under the same conditions, non-protein-bound ferrous iron is oxidized to ferric iron, with the latter only being found in trace detectable levels (10^−18^ mol/L), since it mostly precipitates by forming insoluble polymeric ferric oxyhydroxide complexes with high stability constants (log K = 38) [20]. In general, the solubility of iron and other metals increases at acidic pH levels, including in the acidic environment of the stomach.

## 2. Diseases of Iron Metabolism Imbalance and Their Treatment

Iron imbalance is associated with serious clinical conditions affecting many categories of patients [2,3,20,21]. In particular, iron deficiency anemia (IDA) affects about one-third to one-quarter of the world’s population, with the symptoms including increased child and maternal mortality, pregnancy complications, cardiac complications, fatigue, reduced physical and mental performance and paleness [22,23,24,25]. In many of these cases, the symptoms of iron deficiency are cured using iron supplements, which are widely available in different formulations [26,27,28,29]. In general, however, most of the iron formulations suffer from low specificity, which causes reduced iron absorption and also toxicity in the gastrointestinal tract due to the presence of excess non-absorbed iron [30,31,32]. In many clinical cases such as cancer, kidney disease and other categories of patients, intravenous iron formulations are used, which can also be effective in treating IDA, but toxic side effects have also been reported [33,34,35,36,37,38,39,40]. As in many diseases, a risk/benefit assessment is required for the selection of the appropriate iron formulation and route of administration in each IDA patient case. Additional factors may also be observed in the selection of the iron formulation, such as demographic reasons and costs [41,42].

There are many other diseases related to iron metabolism imbalances in addition to IDA, including the hemoglobinopathies and idiopathic hemochromatosis, which are the most common genetic diseases affecting humans [43,44,45,46,47,48,49]. The hemoglobinopathies include thalassemia and sickle cell disease, which are mainly found in developing countries. It is estimated that about 100,000 children are born annually with thalassemia, mainly in Southeast Asia, the Middle East and Mediterranean countries, and about the same number are born with sickle cell anemia [43,44,45]. For example, thalassemia is endemic in Cyprus, where 1 in 6 persons is an asymptomatic thalassemia heterozygote carrier and about 1 in 1000 is a thalassemia major or thalassemia intermediate patient. The prevention and treatment programs for thalassemia and especially chelation therapy impose a great financial burden to the health budget of many developing countries [43,50,51].

The major form of treatment of thalassemia major is regular red blood cell transfusions every 1–4 weeks and daily chelation therapy [45,51]. Multiple transfusions cause increased iron deposition and damage to the heart, liver, spleen and other organs unless iron chelation therapy is introduced early in life [45,51]. Iron overload in thalassemia has the highest rate of morbidity and mortality of metal-related intoxication in humans [51]. Iron chelation therapy in thalassemia and other transfusional iron loading conditions is carried out worldwide using the generic drugs deferoxamine (DF), deferiprone (L1) and deferasirox (DFRA) [52,53,54,55,56,57]. The combination of chelating drugs, and especially the L1/DF combination, is also widely used in many countries [51,52,53,54,55,56,57].

There are many other diseases related to iron and other metal imbalance and toxicity conditions, where iron-chelating drugs and iron chelator complexes could be used as originally suggested in 2003 [58]. Many of these diseases affect millions of patients, including cancer, Alzheimer’s and Parkinson’s diseases, Freidreich’s disease, AIDS, infectious diseases and aluminum and other metal intoxication conditions [58]. The priority in the selection of diseases for testing the efficacy and toxicity of chelating drugs is based on a risk/benefit assessment in comparison to other available therapeutics in each disease [55]. The repurposing of chelating drugs in non-iron-loaded diseases was facilitated following new scientific evidence and also safety findings, especially related to the number of non-iron-loaded patients and conditions used for clinical trials, which progressively has increased in the last few years [59].

## 3. The Design and Development of Deferiprone and the Maltol–Iron Complex

Deferiprone and the tri-maltol–iron complex, also known as ferric maltol, Feraccru or Accrufer, were originally designed, synthesized and screened in vitro and in vivo in 1981 following the initiation of an academic project in 1978 [60,61,62,63]. Eventually, this project led to the discovery of the novel alpha-ketohydroxypyrone or alpha-hydroxypyrone and alpha-ketohydroxypyridine or alpha-hdroxypyridone class of iron chelators (1978–1981), which were intended for clinical use in diseases related to iron metabolism [60,61,62,63].

The path of development and the journey through the years of each of these drugs were completely different, with many difficulties and many intriguing turns of events related to research findings and the involvement of different academic centers, pharmaceutical companies, physicians, research investigators and patient organizations, which continue today [33,63].

**Figure 1:**
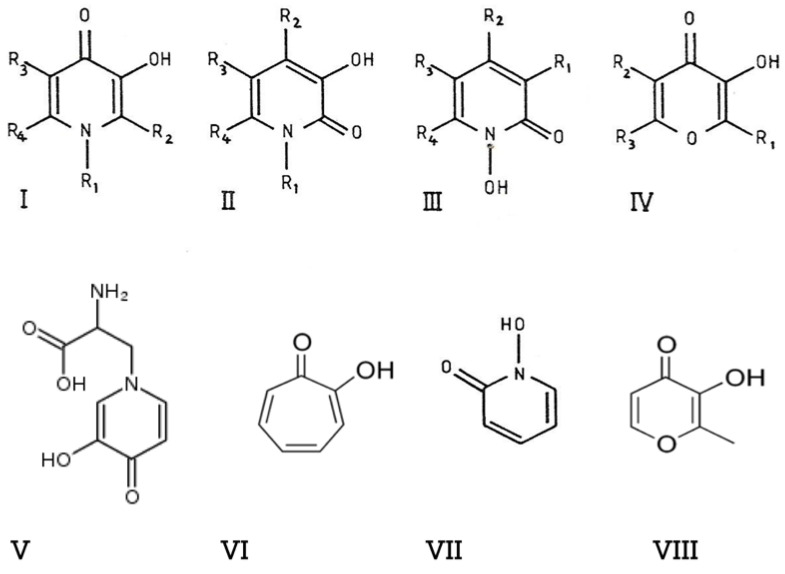
The design of the alpha-ketohydroxypyridine and alpha-ketohydroxypyrone iron chelators for clinical use. The chemical structures of four classes of iron chelators, namely 3-hydroxypyrid-4-ones (**I**), 3-hydroxypyrid-2-ones (**II**), 1-hydroxypyrid-2-ones (**III**) and 3-hydroxypyr-4-ones (**IV**), were identified 40 years ago, following a structure–activity simulation based on the prototype structures of the known compounds mimosine (**V**), tropolone (**VI**), 1-hydroxypyrid-2-one (**VII**) and maltol (**VIII**). Chemical synthesis and iron binding studies led to the development of the drugs deferiprone (L1) and maltol–iron for the treatment of iron overload and iron deficiency anemia, respectively (see [60]).

The design and development of L1 and the maltol–iron complex were initiated in the biological chemistry department of the University of Essex, United Kingdom (UK), while working on a hemoglobin project in 1979 [60,64]. The work on both drugs at this time period was partly supported by the British Technology Group (BTG), a British government organization, and the UK Thalassemia Society (UKTS), a patient charity organization [60,64]. As a result of this academic project, many different groups of iron chelators were initially screened in an in vitro system, with disappointing results until the discovery and identification towards the end of the project of the alpha-ketohydroxy (or alpha-oxohydroxy) metal-binding site on an heteroaromatic ring, which led to the discovery of the novel alpha-ketohydroxypyrone and alpha-ketohydroxypyridine class of iron chelators (1978–1981), which were intended for clinical use in diseases related to iron metabolism [60,61,62,63]. The alpha-ketohydroxy metal-binding site on an heteroaromatic ring was previously reported from natural phytochelator products such as mimosine, maltol and tropolone (Figure 1) [60,61,62,63,64,65].

Based on these prototype structures of alpha-ketohydroxypyrone and alpha-ketohydroxypyridine classes of chelators, about half a dozen new compounds were initially synthesized, including L1, and about a dozen commercially available compounds including maltol were screened in vitro in different models for their iron-binding and other properties [60]. The in vitro screening system included studies on the molecular structure–activity correlation, the air and water stability and solubility of the chelator and its iron complex, the electron-releasing or -withdrawing effects of substituents present on the heteroaromatic ring with regards to iron affinity, the lipid–water partition of the chelator and chelator iron complex and stability of the iron complexes. As a result of the chemical synthesis approach that was used, new chemical synthetic routes were proposed, and also the prospect of the preparation of thousands of new alpha-ketohydroxypyrone and alpha-ketohydroxypyridine compounds with different substituents attached to the heteroaromatic ring was suggested, which could be synthesized and tested using a similar screening system [60] (Figure 1). The synthetic screening proposal included dimeric, trimeric and polymeric chelating drugs, some of which were also synthesized and tested at a later stage [60,66].

Due to lack of different screening facilities at Essex University for investigations related to the chelating compounds, including those for cell and in vivo studies, the developmental program for the iron chelators was partly moved to University College London Medical School (UCLMS) through contacts with the UKTS and associated researchers in UCLMS. In this context, new experimental screening programs were developed and showed that L1 was the most effective chelator for iron mobilization from transferrin and ferritin and also from iron-loaded mice following oral and intraperitoneal administration routes [60]. Similarly, using a different screening program, the maltol–iron complex and other lipophilic iron complexes have been shown to be effective in increasing the transport of iron into red blood cells and inverted rat intestines [60]. The results of these experiments, and especially the effects of L1 and the maltol–iron complex, were not allowed to be published for several years after the discovery, due to BTG “patent” restrictions, to the dismay of the UKTS and other patient organizations.

The situation was particularly urgent and frustrating regarding the development of orally administered L1 in thalassemia, since many patients’ lives were at risk because of complications with the 8–10 h long subcutaneous (sc) administration of DF, including compliance, toxicity and efficacy concerns [45]. In the meantime, another major invention was developed in earlier clinical studies in thalassemia patients supported by the UKTS and initiated at UCLMS using DF suppository formulations, which was published without restrictions [67]. Clinical studies at University College Hospital have shown encouraging preliminary results, with significant increases in urinary iron excretion in thalassemia patients using DF suppository formulations. Furthermore, these clinical studies have also shown the first ever evidence that DF could be absorbed from the rectum, and also that DF was not effective when it was administered orally because of molecular damage in the acid environment of the stomach [67].

### 3.1. The Pre-Clinical and Clinical Development of Deferiprone

The pressure by thalassemia patients and parents was mounting for the rapid development of oral chelation therapy by the early 1980s. As a result, in 1985, the UKTS sponsored the continuation of the iron chelation project at the Royal Free Hospital School of Medicine (RFHSM) of the University of London from where the first publications of the iron removal effects of L1 in comparison to parenteral DF in mice and rabbits and later in other animal species were reported [68,69,70,71,72,73]. Eventually, many other differences, including those found by academics and funding organizations for oral chelating drug selection and development processes, resulted in fierce competition for the promotion by the UCLMS with the BTG of different oral chelators, and especially lipophilic analogues of L1, in contrast to the selection of L1 at the Royal Free Hospital School of Medicine (RFHSM), which was supported by the UKTS for the treatment of iron overload [74,75,76,77].

The higher levels of safety and efficacy of L1 in different animal species in comparison to many other alpha-ketohydroxypyridines studied at the RFHSM, and several other developments such as the simple inexpensive synthesis of L1 and other alpha-ketohydroxypyridines, prompted the initiation of an application to the UK governmental regulatory authorities for clinical testing [78,79]. The application included the physicochemical characteristics and purity of L1, toxicological studies, drug formulation information and a dose protocol, RFHSM ethical committee approval and personal insurance for volunteers and patients participating in the studies, who were financially covered by the UKTS, along with many other requirements.

**Figure 2:**
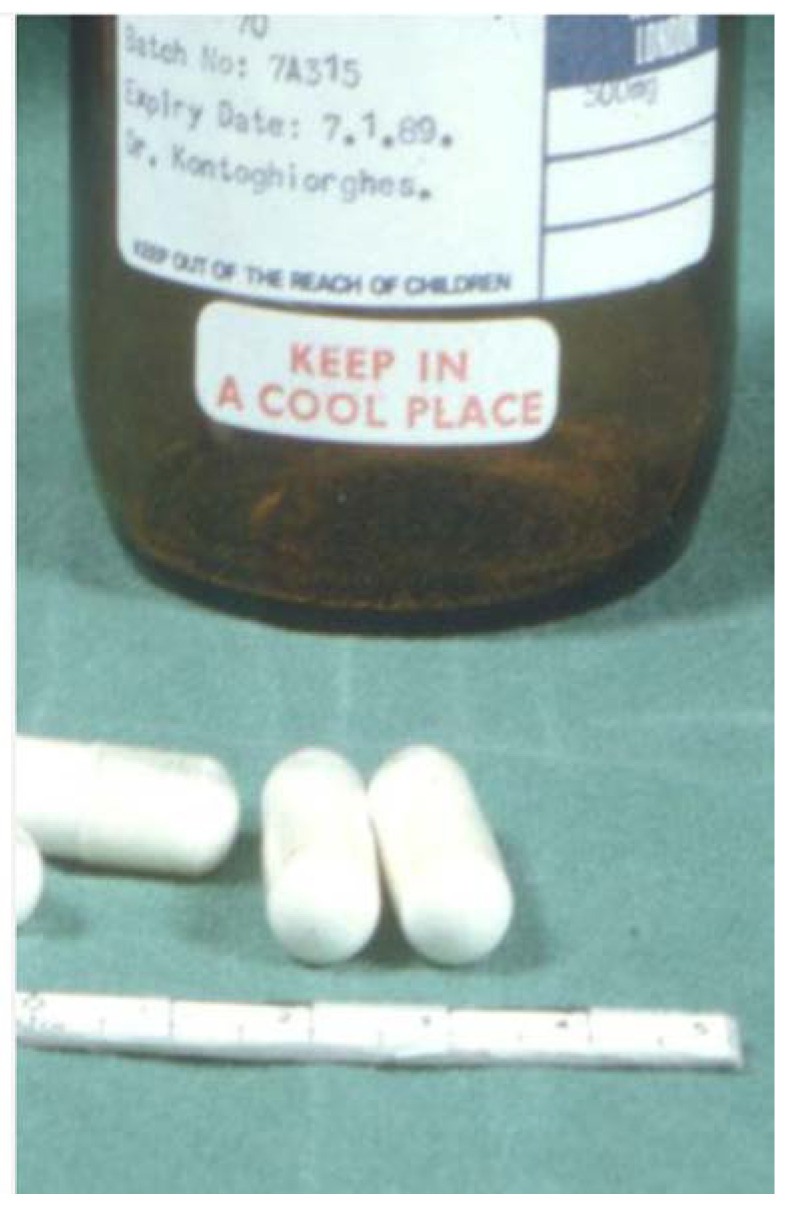
The first pharmaceutical formulation of deferiprone (L1) in transparent gelatin capsules, which were used for the first clinical trials in London, UK, and also in multicenter clinical trials that followed worldwide. Each capsule contained 0.5 g of L1 white solid, with no preservatives or additives (see https://www.youtube.com/watch?v=ZcvSLyIgYd8, accessed on 1 March 2023).

In the meantime, several kilograms of L1 were prepared using the new method of synthesis, which was encapsulated in 0.5 g quantities by the pharmacy of Royal Free Hospital using gelatin capsules (Figure 2). The encapsulation of the white crystalline solid of L1 was necessary because of its bitter taste, which was identified by the inventor [63,78]. Phase I–II clinical trials began using escalating doses of oral L1 (0.5–3.0 g) and iron balance studies in a group of iron-loaded myelodysplacia and thalassemia patients not tolerant to sc DF treatment [80,81]. The results of the clinical studies were encouraging, with increases in urinary iron excretion above the background level, even with low doses of 10 mg/kg, establishing for the first time that iron chelation in thalassemia is possible through the oral route and that L1 was the first oral iron-chelating drug in medicine [82,83]. The drug was well tolerated using different dose protocols, including the maximum dose selected at the time (110 mg/kg/day), which caused equivalent levels of iron excretion to sc DF at equivalent doses, with no increases in other essential metal ion excretion indicators [81].

In the meantime, fierce competition against the development of L1 was initiated by academic and commercial groups, which began from the time of discovery and continues today for different reasons [60,63]. For example, at the initial stages, a lipophilic analogue of L1 namely 1,2-diethyl-3-hydroxypyrid-4-one (EL1NEt) was promoted by different groups of academics and supported by the BTG via studies at Essex University and UCHMS [74,75,76]. However, based on further studies and clinical trials in thalassemia patients at UCHMS, EL1NEt had a lower therapeutic index than L1 and was later abandoned [77,84,85,86]. Similarly, at an earlier stage, Ciba Geigy (now Novartis), the then manufacturers of DF, obtained all pre-clinical and clinical information on L1 from the UKTS-sponsored academic team with the prospect of its rapid development to benefit patients who were not tolerant to sc DF treatment. Following about 3 years of waiting, Ciba Geigy announced that they had carried out different animal toxicity studies with L1 and finally reported that L1 was toxic and that they cannot be involved in its development [87,88]. However, the evaluation methods used for assessing L1’s toxicity were questioned, as well as the available comparative toxicity data for DF, all of which brought into question Ciba Geigy’s assessment methods and conclusions [89,90,91,92,93]. It should be noted that the LD50 of oral L1 in mice is estimated at 2–3 g/kg and intraperitoneally administered L1 in rats at 650 mg/kg [61,77]

Following the initial clinical trials at the RFHSM with L1, many other academic hospitals caring for thalassemia and other categories of transfusional iron-loaded patients worldwide expressed a great interest in joining the clinical trials and testing L1 in their institutions using the same protocol. In most cases, the vast majority of patients selected for the clinical studies were not responding or were intolerant to sc DF therapy. In many of the external clinical studies, L1 was supplied from the RFHSM (Figure 2) to institutions in other countries, e.g., Italy, Switzerland, The Netherlands and Germany, whereas at a later stage in several other countries, e.g., for India, Switzerland, The Netherlands and Canada, L1 was prepared and supplied by local pharmaceutical companies using the published one-step method of chemical synthesis and physicochemical characterization developed at the RFHSM [78].

While the clinical trials with L1 were continuing and expanding worldwide, an incidence of agranulocytosis was reported in 1989 in a Blackfan–Diamond patient at the RFHSM, causing the postponement of the L1 trials at the RFHSM to the dismay of the UKTS’ investigating team [94]. Several other less serious toxic side effects such as neutropenia, joint and musculoskeletal pains and zinc deficiency were also reported in some patients treated with L1 in the RFHSM and other clinical centers [95,96,97]. However, the low incidence of toxic side effects of L1 reported by other clinical centers, and also the presentation of clinical trial results during the first international conference on oral chelation (ICOC) in 1989 at the RFHSM in London by clinical centers testing L1 worldwide, prompted the re-evaluation and the resumption of the clinical trials of L1 at the RFHSM [79,98,99,100,101,102,103,104].

Despite the encouraging pre-clinical and clinical results with L1, there was insufficient interest from major pharmaceutical companies for its commercial development, mainly because of financial considerations and especially the small number of thalassemia patients that require treatment in developed countries. In the meantime, many steps were taken to further advance the development of L1, including an application by the inventor proposing an international non-proprietary (INN) name for L1 in 1991, which resulted in the adoption by the World Health Organization (WHO) of the INN name deferiprone (WHO drug information list 67, volume 2 of 1992).

Unexpectedly, the major breakthrough in the pharmaceutical development of L1 came from India, which played a leading role in the pharmaceutical development and distribution of the drug in developing countries. This development came about following a collaborative project that was initiated between a parent of a thalassemia patient and member of the Thalassemia Society in Mumbay, India, and the UKTS-sponsored group at the RFHSM. The parent also worked at the Indian generic pharmaceutical company Cipla, which was one of the leading generic drug manufacturers in India [63,95,98]. Information provided by the UKTS-sponsored group working at the RFHSM to Cipla led to the chemical and pharmaceutical preparation of L1 and also the initiation of clinical trials in India [63,95,98]. Following the Indian clinical trials and application to the regulatory authorities, the world-first regulatory approval for L1 was obtained in India and L1 became available to Indian thalassemia patients in 1995, and also to many other thalassemia patients in other countries [65,95,98,105,106].

At about the same time as a BBC TV program highlighting the developments in India, the administration of the RFHSM decided to terminate all research on thalassemia supported by the UKTS, including the research on L1 and chelation therapy in general. This was a major drawback in the ongoing projects of L1, which continued elsewhere in the following years. Similarly, the BTG abandoned the development of all other chelating drugs except L1 and licensed the L1 invention to the generic pharmaceutical company Apotex, Canada, which received regulatory approval from the EU authorities for the use of L1 in 1999, followed by many other countries worldwide and also the USA FDA authorities in 2011, following the intervention of the USA Thalassemia Organization [63,105,107].

**Figure 3:**
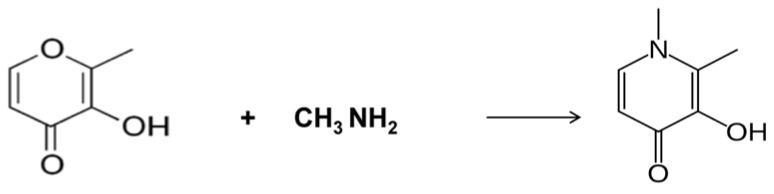
The simple one-step synthesis method of deferiprone (L1) from maltol and methylamine, which was invented in the Royal Free Hospital Medical School. The simple, inexpensive preparation of deferiprone (L1) led to the organization of clinical trials in London, UK, and multicenter clinical trials worldwide. The same synthetic method is currently used by generic pharmaceutical companies worldwide, and most importantly made deferiprone (L1) available to patients in developing countries, where thalassemia and other iron loading diseases are endemic (see [78]).

The academic and commercial conflicts regarding the use of L1 continued following the developmental stage and later introduction of DFRA in 2005 by Novartis, which was also the initial and exclusive manufacturer of DF for many years before the introduction of L1 [108,109,110,111]. It should be noted that for both the generic pharmaceutical companies Cipla and Apotex, L1 was the first non-generic drug to be manufactured and distributed in different countries. The regulatory approval obtained in both cases was similar to that of orphan or emergency drug requirements, which was based largely on published animal and clinical data obtained from the UKTS-sponsored research group at the RFHSM [63]. Deferiprone became a generic drug in 2003 and is still manufactured and supplied to thousands of patients by many generic companies worldwide using the published one-step inexpensive chemical synthesis method (Figure 3) [63,78,112].

The absence of full formal animal or other pre-clinical toxicology studies in the case of L1 by either Cipla or Apotex pharmaceutical companies has put L1 at a “theoretical disadvantage”, and for that reason it was initially classified as a “second-line” iron-chelating drug in comparison to DF and DFRA [68,69,70,71,72,73,77,87,88,89,90,91,92]. However, animal toxicology data for all three drugs have shown that they all are of differential but similar toxicity levels, and in clinical practice L1 is widely used to the same extent as the other two chelating drugs in iron-loaded patients. Most importantly, L1 is regarded as the first-line iron-chelating drug in many categories of patients because of its unique properties, and especially its superior ability to remove excess cardiac iron, as shown by T2* MRI results, the increased long-term survival of transfusional iron-loaded patients and also because of its safety in patients with low iron loads [53,113,114,115,116,117,118,119]. The safety and efficacy of L1, DF and DFRA in iron-loaded patients have been recently reviewed [120]. All three iron-chelating drugs are now generic and widely used in the treatment of iron overload [121]. There is no general consensus on the use of L1, DF and DFRA or of their combinations, as well as the related protocols in transfusional iron-loaded patients. In most cases, the selection of the chelation therapy and related protocols is generally random and based on subjective criteria and with non-specific aims, and as a result their clinical application vary from country to country and from clinic to clinic [51,113].

With regards to its safety, long-term studies and continuous clinical monitoring involving thousands of thalassemia and other categories of patients for the last 30 years have confirmed the low toxicity levels of L1 [97,120]. The most serious toxic side effects of L1 reported in the last 30 years have included reversible agranulocytosis (<1%) and neutropenia (<5%), while the less serious toxic side effects have included gastric intolerance, joint pains and zinc deficiency [97,120]. Toxicity vigilance and prophylactic measures, such as weekly or fortnightly blood counts, are important factors for ensuring the safety of L1 during long-term use. Similarly, the use of zinc supplements for prophylaxis in patients on long-term treatment with L1 and DF is also recommended [120,121].

**Figure 4:**
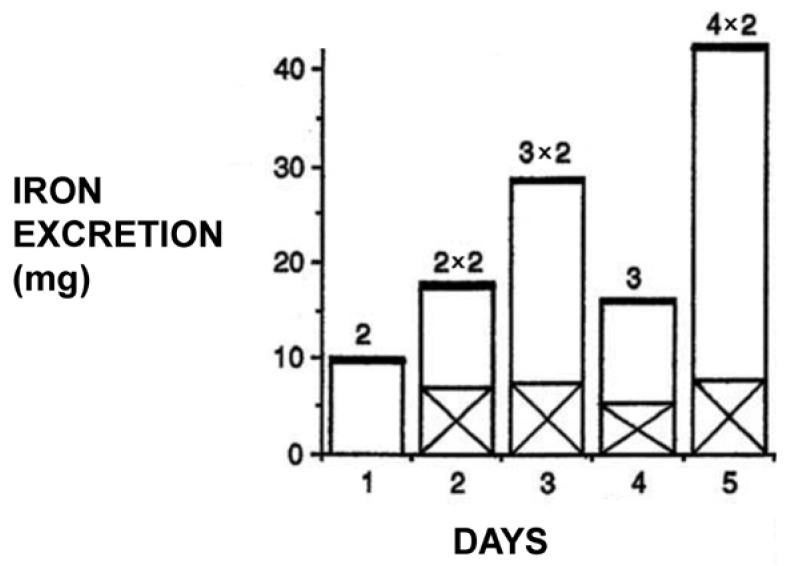
Iron excretion profile in a thalassemia major patient following the daily administration of different doses of oral deferiprone (L1). Iron excretion (mg) in the urine is shown in the open columns and fecal iron excretion in the x columns. Iron excretion is dose-dependent, as shown from the doses (g) of deferiprone (L1) at the top of the column. It should be noted that almost all excess iron is excreted in the urine (see [81]).

Deferiprone’s ability as a monotherapy or in combination with DF to clear all excess iron and maintain normal iron stores using personalized dose protocols in regularly transfused thalassemia major patients signifies the complete treatment of iron overload and the achievement of the major aim of iron chelation therapy (Figure 4) [119,120,121,122,123]. In addition, the continuous worldwide use of L1 in thalassemia and other iron-loaded patients 40 years after its invention and its long-term cardioprotective life-saving effects and other pharmacological properties have changed the prognosis of thalassemia major patients, and in general thalassemia is no longer considered fatal but rather a chronic disease [51,63,119,120,121,122,123].

### 3.2. The Pre-Clinical and Clinical Development of the Maltol–Iron Complex

Unlike the discovery and development of deferiprone, which was regarded as a life-saving drug for thousands of iron-loaded patients, especially in developing countries, the development of the maltol–iron complex was not urgently pursued by pharmaceutical companies. The delay was caused by commercial considerations and especially the availability of a large number of competing iron formulations used for the treatment of IDA, a condition generally considered non-life-threatening [23,26].

The discovery of the maltol–iron complex and its proposed use in IDA originated from the identification in 1979–1982 of the naturally occurring plant product and phytochelator maltol as an alpha-ketohydroxypyrone chelator, which was widely available and used in humans [60]. The selection of the maltol–iron complex for IDA was based on its lipophilic properties and the characterization of many other in vitro and in vivo properties, including the stability of the iron complex, the protein interactions and the ability to transfer iron in red blood cells and inverted intestinal sacs [23,60,65]. Further development of the maltol–iron complex in animal and also clinical studies continued intermittently for many years and by different groups of investigators following its discovery [60,65]. Many other studies are still ongoing for different categories of patients with IDA conditions [65].

Further developmental studies involving the in vivo confirmation of the effects of the maltol–iron complex in relation to IDA were conducted following the original studies and design phase of 1979–1982 [60]. These involved a comparative study in mice using a new screening system of the intragastric administration of different chelator iron (59-Fe) complexes and the monitoring of 59-Fe’s uptake and distribution in the body, as well as its excretion, thereby demonstrating the need for a lipophilic iron complex for IDA [60,124]. In this context, the effect of 16 natural and synthetic chelators on iron (59-Fe) absorption, in addition to maltol, have been investigated in three different experiments using single and repeated intragastric administrations of chelator iron (59-Fe) complexes [124]. Maltol and 2-hydroxy-4-methoxypyridine-1-oxide (L6), both of which form neutral, lipophilic iron complexes, were found to cause significant increases in 59-Fe absorption, while hydrophilic chelators, including DF, L1, mimosine and EDTA, caused significant decreases in iron (59-Fe) absorption in comparison to the controls. Most of the iron (59-Fe) absorbed from the chelator iron (59-Fe) mixtures was detected in red blood cells and less so in the organs, where it was mainly distributed in the liver (50–60%) as compared to the spleen (30–40%) and other organs. These findings have suggested that the iron (59-Fe) absorbed following the oral administration of chelator iron (59-Fe) complexes is diverted primarily to the bone marrow and utilized for the production of hemoglobin [50,124].

In a set of different experiments in rats, increased iron (59-Fe) uptake was observed following the intraduodenal administration of the iron (59-Fe) maltol in comparison to other hydrophilic iron (59-Fe) mixtures with fumarate, gluconate and EDTA [125]. The oral administration and dissociation of iron (59-Fe) from maltol were suggested following its entry into the intestinal wall, with the iron and maltol molecules following separate and different metabolic pathways. In this context, iron from the iron–maltol complex enters the iron metabolism pathway, involving intracellular storage in ferritin, transport in the blood by transferrin and utilization by the hemopoietic tissues for the production of hemoglobin. In the case of dissociated maltol, the metabolic pathway mostly involves glucuronidation in the liver and the formation of maltol glucorunide, which is excreted in the urine [33]. Similarly, the dissociation of maltol and iron was observed following the intravenous administration of an iron (59-Fe)–maltol mixture in rats, presumably through the donation of iron to transferrin and release of maltol in the blood stream [126]. In addition, iron (59-Fe) uptake in rats was mostly associated with hemoglobin production and less so with liver deposition and storage. The overall increased uptake of (59-Fe) iron in the presence of maltol and its utilization by the hemopoietic tissues has been observed in both mice and rats, despite the suggested dissociation of iron and maltol and the kinetic variation in iron uptake rates following the use of different iron concentrations in the mixture [124,125,126].

**Figure 5:**
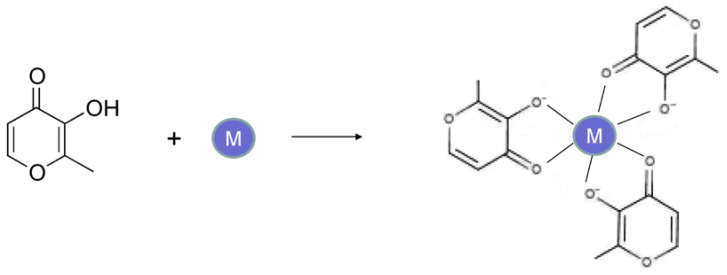
The chemical structure and formation reaction of metal ion complexes of maltol. An octahedral metal ion complex of neutral charge is formed at physiological pH between maltol and trivalent metal (M) ions such as iron, gallium, aluminum and indium. Similar trivalent neutral metal ion complexes are formed using deferiprone (L1).

Despite some initial preliminary interest about the development and commercialization of the maltol–iron complex for IDA by pharmaceutical companies, further animal and clinical studies, including long-term toxicology studies, were not pursued, probably because of the overall expenditure required for a new drug’s development at the time. No further efforts for the pharmaceutical development of the maltol–iron complex were undertaken, and the interest was focused on the pharmacological research aspects of maltol and its iron complex and other metal complexes (Figure 5) [33,60,127]. However, as in many other cases of recent drug development and the introduction of new regulatory approval procedures, a loophole was used whereby the maltol–iron complex was further developed and marketed through the relaxed requirements involving an orphan drug procedure [59]. In this context, several clinical trials of the maltol–iron complex in iron-deficient patients with inflammatory bowel disease and similar diseases were initiated, considering that no effective treatments of IDA were available with other iron formulations for these categories of patients [33].

Many categories of patients have received the maltol–iron complex in a number of clinical studies, including for Crohn’s disease, ulcerative colitis, chronic kidney disease, pulmonary hypertension and also patients intolerant to other iron formulations [128,129,130,131,132,133,134,135,136,137,138,139]. Similarly, the maltol–iron complex was used in clinical pharmacokinetic and comparative studies with other iron formulations. The clinical and pharmacological effects of the maltol–iron complex and other metal complexes were recently reviewed (Figure 5) [33,127]. In general, the maltol–iron complex containing at least 30 mg iron could be effective if taken for a week to three months (twice daily, before breakfast and the evening meal). In such clinical cases, the serum iron, transferrin saturation, serum ferritin and reticulocyte hemoglobin contents increase significantly within a week to higher levels in comparison to other ferric formulations and are also equivalent to those of the widely used ferrous sulfate formulations. Furthermore, the level of iron absorption appears to be proportional to the dose levels of maltol–iron, and despite iron being utilized for hemoglobin synthesis and storage, maltol is cleared from the body, with no sign of accumulation in any in the patients of the different categories that have received the maltol–iron formulation [128,129,130,131,132,133,134,135,136,137,138,139].

There are several other advantages in the use of the maltol–iron complex over other iron formulations, including rapid iron absorption, low toxicity and higher selectivity in iron delivery in comparison to other iron formulations. These advantageous results, cause a decrease in the toxicity associated with the excess non-absorbable iron, which is usually observed with other iron formulations. In addition, the efficacy and low toxicity of the maltol–iron complex could benefit many patients experiencing complications and toxicity with other iron formulations, including intravenous iron and ferrous sulphate [30,36,37,38,130,131,140,141,142,143].

The level of safety of the maltol–iron complex appears in general to be satisfactory. This observation has also been reported in almost all of the clinical studies and trials, whereby the maltol–iron complex has been shown to be tolerable and effective, with significant improvements in anemia for the different categories of patients treated. In this context, the most common adverse effect during the clinical studies was mild gastrointestinal intolerance [130,131].

Overall, the maltol–iron complex may have many general advantages over other iron formulations for the treatment of IDA, which include the ease of administration, low toxicity, short timing for achieving the normalization of hemoglobin levels and low cost. In particular, the low cost of maltol–iron formulations and their general availability are major concerns affecting global health, considering that the vast majority of IDA patients live in developing countries with low health budgets. Furthermore, the low costs of maltol and iron may encourage the development of many different maltol–iron formulations, which could overturn any proprietary and other claims similar to generic pharmaceuticals [59,144,145,146]. Due to the increased availability of low-cost maltol–iron complex formulations, they have prospects of becoming the first option for the worldwide treatment of iron-deficient patients.

## 4. New Clinical Applications of Deferiprone and the Maltol–Iron Complex

While the major efforts in the development of L1 and the maltol–iron were based on the treatment of transfusional iron overload and IDA, respectively, their possible applications in other diseases were also suggested and tested at the early stages of their development, thereby contributing important information in the overall pharmacology and toxicology of both of these two drugs.

The additional fields of investigation related to these two drugs were broadly based on other diseases of iron metabolism, infectious diseases, conditions related to free radical pathology and metal toxicity conditions other than for iron. In the meantime, many other analogues of L1 and iron–maltol were synthesized and tested for comparison with the original findings. The investigations included in vitro, in vivo and clinical studies.

### 4.1. The Use of Deferiprone in Transfusional Iron Overload and Other Diseases

Thousands of patients suffering from iron overload and also other illnesses, as well as normal volunteers, have been treated in the last 40 years with L1, which is regarded as one of the safest drugs in the world per dose, e.g., 50–100 mg/kg received on a daily basis. In this context, the major category of patients receiving L1 is β-thalassemia major patients, followed by many other categories of iron-loaded patients, including β-thalassemia intermedia, HbE β-thalassemia, HbS β-thalassemia, sickle cell anemia, myelodysplastic syndrome, aplastic anemia, Fanconi’s anemia, Blackfan–Diamond anemia, pyruvate kinase deficiency, idiopathic hemochromatosis, iron overload in hemodialysis, and juvenile hemochromatosis [51,53,54,104,147,148,149,150,151,152,153,154].

Similarly, many other categories of patients with normal iron stores have also been receiving L1, mostly in short- but also in long-term clinical trials. The repurposing of L1 for diseases other than iron overload was organized based on similar dose protocols to those of iron overload and took place originally in the in the UK (RFHSM) but also other countries within a few years of initiating the clinical trials with transfusional iron-loaded thalassemia and myelodysplasia patients [80,81,82,83]. The clinical use of L1 in several other categories of patients with normal iron stores has increased with time and involved many other different dose protocols, durations of administration and numbers of patients in each category. The decision to use L1 in these conditions was in most cases related to the absence of other effective therapies. Some of the categories of diseases reported to have received L1 include renal dialysis, aluminum overload, Friedreich’s ataxia, Parkinson’s and Alzheimer’s diseases, neurodegeneration with brain iron accumulation, pantothenate kinase 2-associated neurodegeneration (PKAN), rheumatoid arthritis, aceruloplasminemia, glomerulonephritis and diabetic nephropathy, malaria and HIV [155,156,157,158,159,160,161,162,163,164,165,166].

In general, no serious toxicity was reported for clinical trials of up to 9 months duration and with a maximum dose of 100 mg/kg/day in patients with normal iron stores. Most importantly, significant clinical improvements have been reported in most of these categories of patients [155,156,157,158,159,160,161,162,163,164,165,166]. Positive outcomes have also been reported, where L1 was used in adjuvant and combination therapies, which was similar to iron-loaded cases [164,167].

**Figure 6:**
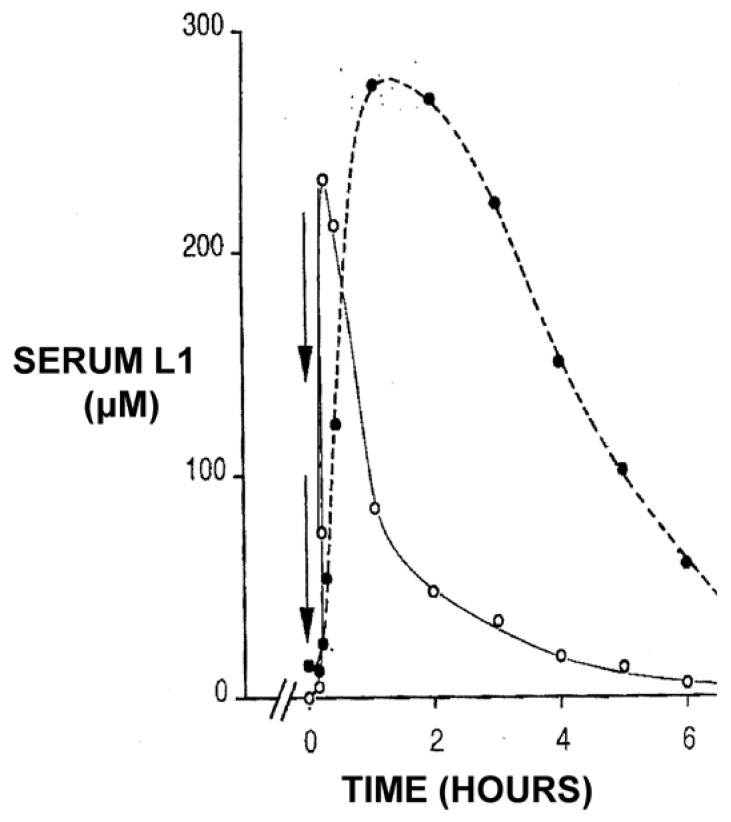
Pharmacokinetic profile of deferiprone (L1) and its glucuronide metabolite conjugate following its oral administration in a thalassemia patient. Deferiprone (L1) 3 g was administered at time 0, which was rapidly absorbed (open circles), peaked within one hour and cleared from the blood in about 6 h. The blood clearance rate of the glucuronide metabolite conjugate (black circles) was slower, as monitored by measuring the absorbance at 280 nm (see [107,170]).

**Figure 7:**
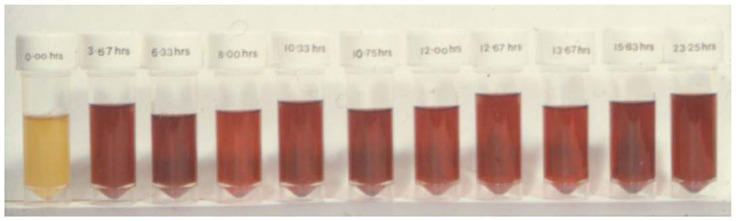
The sequential urinary iron excretion profile following intensive chelation therapy with deferiprone (L1) in a heavily iron-loaded thalassemia patient. Divided doses of L1 to a total of 250 mg/kg were administered in the thalassemia patient and sequential urine samples were collected over 24 h, totaling 325 mg of iron (see [169]).

However, it is unfortunate that in many of the recent clinical trials involving mainly neurodegenerative disease patients, no rationale for the selection of the L1 dose protocol nor of iron metabolism balance studies was given [158,159,160]. For example, in many such studies, L1 was used in single or repeated doses of 15 mg/kg/day with disappointing results [158,159,160]. It should be noted that previous dose escalation and iron metabolism balance studies have shown that the use of such low doses of L1 in iron-loaded and non-iron-loaded conditions was mostly ineffective in increasing iron excretion or the improvement of other hematological parameters [80,81,167,168]. The selection of posology is critical for evaluating the risk/benefit assessment of L1 and all other drugs. Furthermore, the rationale for the dose protocol selection for the use of L1 and all other drugs in any disease should be accompanied by pharmacological and therapeutic evidence such as the estimation of the pharmacokinetic, metabolic and other parameters and the critical drug concentration for optimal therapeutic activity, as previously shown for the use of L1 in thalassemia patients (Figure 6 and Figure 7) [80,81,169,170].

It can be suggested in general that the positive results in terms of safety and the diverse categories of iron-loaded and other patients treated in clinical trials with L1 have increased the prospects of the wider use of the drug in many other diseases, including diseases related to free radical pathology and also in cancer [171,172,173].

### 4.2. The Use of Maltol–Metal Complexes in Diseases Other Than Iron Deficiency Anemia

The identification of the possible applications of maltol and the maltol–iron complex in medicine was proposed following their physicochemical characterization, protein and cellular interactions and in vivo and other studies [60]. Within this context, many investigations have been carried out on the possibility of the clinical use of metal complexes of maltol other than iron, such as zinc complexes as supplements for zinc deficiency, maltol–gallium complexes for the treatment of cancer, maltol complexes with diagnostic metals such as indium for use in clinical diagnosis and maltol complexes with theranostic metals such as (68-Ga) gallate (Figure 5) [33,60].

The maltol–gallium complex (gallium maltolate or maltol gallate) has attracted a lot of interest because it has reached the stage of clinical trials and veterinary use, mainly for its anticancer and antimicrobial activities [174,175,176,177,178,179,180,181]. The basic mode of action in relation to the anticancer and antimicrobial targeting of the maltol–gallium complex is the disruption of iron metabolism pathways by mimicking iron. In particular, the antimicrobial activity of the maltol–gallium complex is thought to cause the partial deprivation of iron, which is essential for the rapid growth of pathogenic microbes [178,179,180,181,182,183,184]. Similarly, the anticancer activity of the maltol–gallium complex is thought to be based on the delivery of gallium to transferrin, causing a reduction in iron uptake by the cancer cells and a reduction in their growth through the slow turnover of the iron-dependent enzyme ribonucleotide reductase, resulting in the inhibition of DNA synthesis [19,185,186,187]. This mechanism of cancer cell inhibition mainly affects cancer types such as breast, prostate and bladder cancers and leukemias, which involve an abundance of transferrin receptors and the upregulated production of ribonucleotide reductase [18,19,185,186,187,188]. A further mechanism of DNA inhibition by the maltol–gallium complex is thought to involve the transfer and incorporation or binding of gallium to nucleotide substrates and subsequently reduced DNA synthesis [19].

Another major area of application to medicine in relation to iron and other metal complexes of maltol is their use in the theranostic and diagnostic fields, which are rapidly expanding, involving many diseases [19,189,190,191,192]. The maltol complexes involving different metal ions have variable physicochemical and biochemical properties, which appear to affect the bodily distribution of the metal ions and radiotracers, similar to other metal chelator complexes [19,189,190,191,192,193,194,195]. Targeted chelator metal complexes have been identified for the diagnostic and theranostic application of radiotracer metals in cancer, inflammation and other diseases (Figure 5) [19,193,194,195]. In clinical studies in hepatocellular carcinoma patients, for example, cancer cells have been found to be highly gallium-avid in (67)Ga diagnostic scans. In this context, treatment with an oral maltol–gallium complex caused significant improvements of cancer indices and necrosis of the tumor [175].

The increased number of applications of maltol–metal complexes in medicine and veterinary medicine, as well as the pharmacological and toxicological information obtained from relevant studies, has increased the prospect of their wider use in many other fields of diagnostic and theranostic medicine [196,197,198,199]

## 5. Future Prospects in the Use of Deferiprone and the Maltol–Iron Complex

There are increasing prospects for the wider application of L1 and the maltol–iron complex in medicine, including for the optimization of existing therapeutic applications involving improved dose protocols, as well as combinations with other drugs or natural products, personalized medicine adjustments, the treatment of more and different clinical conditions and also more diagnostic and theranostic applications. In particular, there are many metabolic pathways and associated diseases that can further be investigated and targeted, with some involving iron interactions and free radical pathologies, many diseases related to metal metabolic pathways and toxicity other than iron, metal supplementation in essential metal ion deficiency diseases and new drug formulations and routes of administration. Many of these new applications have been suggested from relevant in vitro and in vivo studies and also following new clinical evidence [20,33,63,173,186].

**Figure 8:**
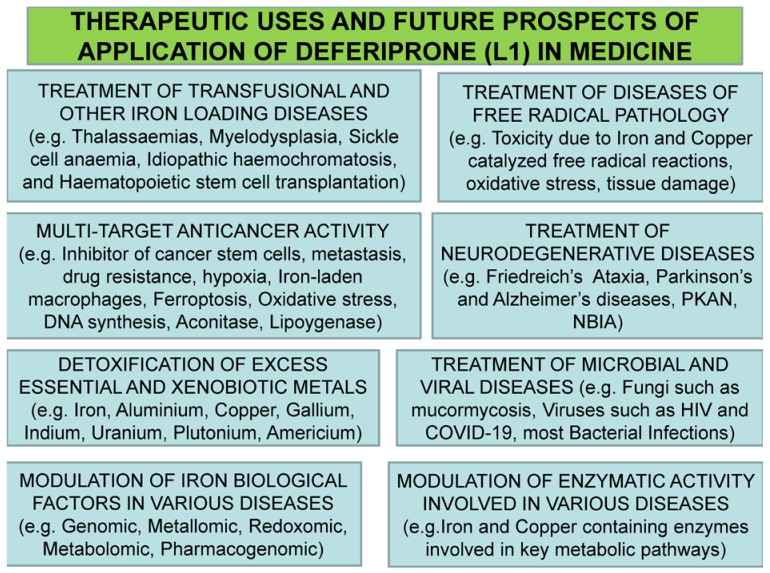
Ongoing therapeutic uses and future investigations of deferiprone (L1) in medicine. Deferiprone (L1) is widely used in the treatment of iron overload worldwide and also other conditions where the present treatments appear to be ineffective. The wide range of therapeutic application prospects is related to its safety profile and also the access and distribution in almost all organs at therapeutic concentrations. PKAN: pantothenate-kinase-associated neurodegeneration; NBIA: neurodegeneration with brain iron accumulation.

The emphasis on new clinical applications of many drugs, including both L1 and the maltol–iron complex, is usually related to orphan diseases and diseases with no effective treatments [59]. In this context, an important application for L1 and the maltol–metal complexes is the anticancer activity of newly identified cancer targets and metabolic pathways in one or more cancer types, which are in addition and not directly related to previously established and discussed targets (Figure 8) [59,173,186].

Examples of previously established cancer targets include the classical restriction or reduction of the iron supply, inhibition of transferrin iron delivery and inhibition of ribonucleotide reductase in DNA synthesis, the high antioxidant potential by inhibiting free radical formation etc., [18,173,182,186,200,201,202,203,204,205,206,207]. The new anticancer strategies involving the new anticancer targets for L1 include the modulation of ferroptosis, ferritin iron removal and the control of hyperferritinemia, the inhibition of hypoxia related to the role of hypoxia-inducible factor (HIF), the modulation of the function of new molecular species such as the metalloreductase “six transmembrane epithelial antigen of prostate, family member 4 protein” (STEAP4) and the metastasis suppressor N-MYC downstream-regulated gene-1 (NDRG1), the modulation of metabolic pathways of oxidative stress damage affecting mitochondrial function and the removal of the excess iron from iron-laden macrophages [173,208,209,210,211,212,213,214,215,216,217,218,219,220,221]. In general, these iron metabolism pathways and other targets appear to affect all stages and types of cancer progression and to be modulated by L1, which was suggested as one of the most potent EMA- and FDA-approved drugs for targeting cancer stem cells and a potential anticancer drug for breast, prostate, glioblastoma and other cancers [173,222,223,224].

**Figure 9:**
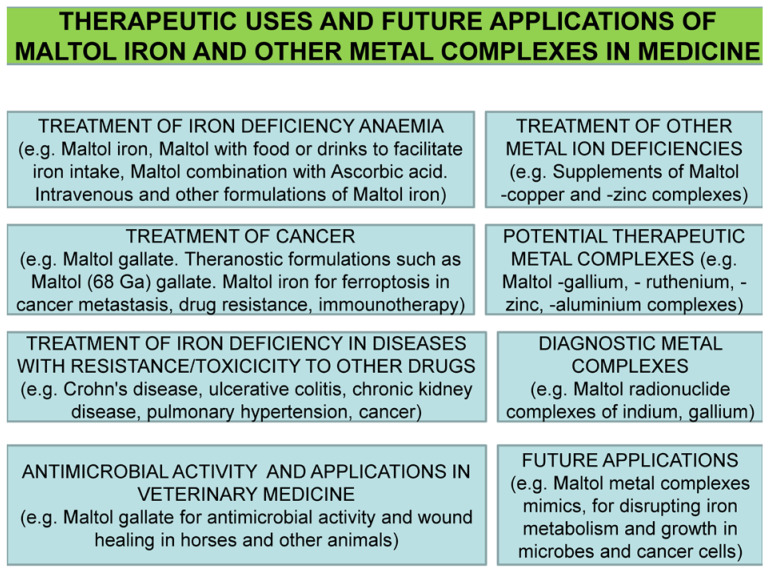
Ongoing therapeutic uses of maltol–iron in iron deficiency anemia and future applications of maltol–metal complexes in other fields of medicine. Many categories of patients may benefit from iron and other maltol–metal complexes. The wide range of therapeutic application prospects is related to the safety profile of the maltol–metal complexes.

In contrast to the mode of action of L1 in the above targets, lipophilic chelator metal complexes similar to maltol–iron and also other more lipophilic iron complexes have been proposed for the induction of ferroptosis in cancer cells, especially in cases of refractory or recurring tumors, and also in cases of drug resistance and metastasis (Figure 9) [173,188,204]. It should be noted that about 90% of the cancer patients develop resistance to existing drugs, and also that about 90% of the mortality in cancer patients is related to metastasis [173,225,226]. Overall, opposing modes of action of anticancer activity are shown by L1 and lipophilic iron complexes, which can be utilized for targeting different stages and types of cancer [173,205,227,228,229].

The prospect of the application of L1 in infectious, inflammatory and many other diseases in addition to cancer has been previously reviewed [19,20,183,184]. However, new findings and related concepts are developing, which constitute new pharmacological targets for L1 and other chelators or chelator iron complexes, including maltol–iron. For example, common targets for chelators are hyperferritinemia and iron-laden macrophages, which are found in cancer and inflammatory and infectious diseases, including COVID-19 [173,230]. In particular, with regards to the modes of action of L1 in COVID-19, these include antiviral, antimicrobial, antioxidant, antihypoxic and antiferroptotic effects, as well as iron-mobilizing effects from ferritin, macrophages and other cells involved in the immune response and hyperinflammation. Some of the pharmacological and other characteristics of L1, including its extensive tissue distribution, could also facilitate its use in the treatment of COVID-19 through drug combinations, adjuvant therapies and disease prevention.

In general, the characteristics of the targets can impose many limitations in the selection of therapeutics, including chelating drugs for the optimization of treatments. For example, only L1 from all three iron-chelating drugs (L1, DF, DFRA) can cross the blood–brain barrier and target infections in the brain, such as meningitis caused by *Neisseria meningitides* or cancer types such as glioblastoma, both of which depend on iron supply for growth [19,20,173].

There are many other areas of potential development and applications of L1 and maltol–iron complexes. For example, intravenous L1 and maltol–iron complex formulations may have to be designed to benefit cancer and other categories of patients unable to receive oral formulations [34,40,185,231,232,233,234,235]. Similarly, combinations with other drugs may enhance the therapeutic efficacy in comparison to monotherapies in many diseases [81,234,235,236]. This was shown in the treatment of iron overload in thalassemia using the ICOC L1/DF combination, and similar effects are expected in terms of gastrointestinal iron uptake for the treatment of IDA using mixed chelator iron complexes of maltol and ascorbate [33,81,234,235,236,237,238,239]. Further developments are expected from the use of other metal complexes, which may result in the improvement of the diagnostic and theranostic applications, as well as in synergistic effects with other drugs or proteins in the treatment of different diseases [20,33,240,241,242,243,244,245,246,247,248,249,250,251,252,253,254,255,256,257,258,259]. In contrast, L1 can be used for the detoxification of many xenobiotic metals, including those used in diagnostic and theranostic applications [195,260,261,262]. Furthermore, improved formulations may increase the efficacy and decrease the toxicity of L1 and the maltol–iron complex in different clinical conditions [263]. In each of the above-described cases, specific conditions must be applied and specific protocols need to be designed to achieve optimal therapeutic activity and effects.

## 6. Conclusions

Despite the many difficulties and controversies, L1 and the maltol–iron complex are prime examples of academic drug discovery and orphan drug development medicinal products. In particular, L1 has helped in the treatment and survival of hundreds of thousands of iron-loaded and other patients worldwide in the past 30 years. Similar results are expected from the use of the recently approved maltol–iron complex for the treatment of IDA, which affects 1 in 3 or 4 individuals worldwide.

The repurposing of L1 in many categories of non-iron-loaded patients, including neurodegenerative, infectious, inflammatory, metal toxicity and other conditions, signifies the safety and multitargeting potential of the drug. Similarly, the use of the maltol–gallium complex in cancer and also of other maltol–metal complexes in other therapeutic, theranostic and diagnostic applications highlights the future routes of development and use of new therapeutic, theranostic and diagnostic maltol and other chelator metal complexes.

New advancements in the pathology of free radicals, cancers and many other diseases, including the discovery of new iron and other metal metabolic routes and proteins that can be modulated by L1, maltol–iron and also other new chelators and metal complexes, underlines the importance of chelators and chelator metal complexes in medicine. In particular, their use as monotherapies or in combinations with other drugs is essential for the improvement of the treatment of many diseases and increased prospects of patients’ survival, especially in many diseases with no effective therapies.

## Data Availability

Not applicable.

## Abbreviations

EMA	European Medicines Agency
COVID-19	coronavirus disease 2019
DF	deferoxamine
DFRA	deferasirox
EDTA	ethylenediaminetriacetic acid
FDA	Food and Drug Administration (USA)
HIF	Hypoxia-inducible factor
ICOC	International Committee on Chelation
IDA	iron deficiency anemia
L1	deferiprone
MRI	magnetic resonance imaging
NDRG	1N-MYC downstream-regulated gene-1
NBIA	neurodegeneration with brain iron accumulation
PKAN	Pantothenate-kinase-associated neurodegeneration
STEAP4	six transmembrane epithelial antigen of prostate, family member 4 protein
